# The Functional Polymorphism of *DDAH2* rs9267551 Is an Independent Determinant of Arterial Stiffness

**DOI:** 10.3389/fcvm.2021.811431

**Published:** 2022-01-03

**Authors:** Carolina Averta, Elettra Mancuso, Rosangela Spiga, Sofia Miceli, Elena Succurro, Teresa Vanessa Fiorentino, Maria Perticone, Gaia Chiara Mannino, Prapaporn Jungtrakoon Thamtarana, Angela Sciacqua, Giorgio Sesti, Francesco Andreozzi

**Affiliations:** ^1^Department of Medical and Surgical Sciences, University Magna Graecia of Catanzaro, Catanzaro, Italy; ^2^Research Center for the Prevention and Treatment of Metabolic Diseases (CR METDIS), University Magna Graecia of Catanzaro, Catanzaro, Italy; ^3^Siriraj Center of Research Excellence for Diabetes and Obesity, Division of Molecular Medicine, Department of Research, Faculty of Medicine Siriraj Hospital, Mahidol University, Bangkok, Thailand; ^4^Department of Clinical and Molecular Medicine, University of Rome-Sapienza, Rome, Italy

**Keywords:** pulse wave velocity, arterial stiffness, dimethylarginine dimethylaminohydrolase, rs9267551, ADMA

## Abstract

**Background:** The association of circulating asymmetric dimethylarginine (ADMA) levels with cardiovascular risk and arterial stiffness has been reportedly demonstrated, although the causal involvement of ADMA in the pathogenesis of these conditions is still debated. Dimethylaminohydrolase 2 (DDAH2) is the enzyme responsible for ADMA hydrolysis in the vasculature, and carriers of the polymorphism rs9267551 C in the 5′-UTR of *DDAH2* have been reported to have higher *DDAH2* expression and reduced levels of serum ADMA.

**Approach and Results:** We genotyped rs9267551 in 633 adults of European ancestry and measured their carotid–femoral pulse wave velocity (cfPWV), the gold-standard method to estimate arterial stiffness. cfPWV resulted significantly lower in rs9267551 C allele carriers (Δ = −1.12 m/s, *P* < 0.01) after correction for age, sex and BMI, and a univariate regression showed that the presence of rs9267551 C variant was negatively associated with cfPWV (β = −0.110, *P* < 0.01). In a multivariable regression model, subjects carrying the rs9267551 C allele manifested significantly lower cfPWV than GG carriers (β = −0.098, *P* = 0.01) independently from several potential confounders. We measured circulating ADMA levels in a subset of 344 subjects. A mediation analysis revealed that the effect of *DDAH2* rs9267551 genotype on cfPWV was mediated by the variation in ADMA levels.

**Conclusions:** These evidences hint that the presence of rs9267551 C allele may explain, at least in part, a reduction in vessel rigidity as measured by cfPWV, and support the attribution of a causative role to ADMA in the pathogenesis of arterial stiffness.

## Introduction

The arterial wall is characterized by its high elasticity, which participates to the physiological regulation of this highly pressurized compartment. The stiffness of the arterial wall increases gradually from the central segments to the periphery, determining a gradient of blood pressure, and although brachial blood pressure is commonly measured in clinical practice, aortic blood pressure correlates more strictly with markers of cardiovascular risk (1). An augmented rigidity of the vessels is associated with increased occurrence of cardiovascular disease (2), stroke (3) and kidney disease (4). Several factors contribute to the stiffening of arterial walls as a normal consequence of aging, but arterial elasticity may also be found reduced in younger subjects who carry cardiovascular risk factors such as hypertension, obesity and dyslipidemia. The latter phenomenon suggests that exposure of the vessels to the milieu determined by those pathologic conditions may interfere with the balance of hemodynamic forces (5).

Pulse wave velocity (PWV) is a simple measurement representing the average stiffness between two sites of the arterial system, with higher values indicating the presence of higher resistance (stiffer vessels), and it is considered as a *bona fide* surrogate early marker of atherosclerosis and cardiovascular morbidity and mortality (6, 7). Indeed, several guidelines propose carotid–femoral PWV (cfPWV), the current gold-standard method used to evaluate arterial stiffness, as a conservative method to determine the presence of aortic function alteration (8–10), and it is an independent predictor of cardiovascular events and all-cause mortality in the general population and in high-risk subjects (2, 11–13).

In the subclinical phases of the atherogenic process the reduced availability of the vasodilator hormone nitric oxide (NO) may foster endothelial dysfunction. The endothelial NO synthase (eNOS) is the enzyme responsible for the synthesis of NO in the vascular district, starting from the substrate L-arginine. Asymmetric dimethylarginine (ADMA) is an endogenous methylated form of arginine able to competing with L-arginine and to inhibit NO production (14, 15). Several research efforts have demonstrated the existence of an association between elevated circulating levels of ADMA and major cardiovascular events or mortality (16–20). In spite of this, the predictive role of ADMA is still subject of debate (21–24). Altogether, there is an evidence gap regarding the causal role of ADMA in the dysregulation of arterial blood pressure and endothelial function. In 2006 a small intervention trial demonstrated that acute exogenous administration of ADMA in healthy male volunteers was able to induce a reduction of cerebral blood flow and arterial compliance due to increased arterial stiffness (25). Very recently, Malle et al. (26) have attempted to resolve this gap and they could not detect the presence in hypertensive patients of a significant association between ADMA and blood pressure or PWV. It is worth noticing, though, that observational studies cannot rule out the masking influence of confounding factors, i.e., due to co-morbidities, or establish whether a marker is the cause or the manifestation of a specific condition, due to their non-interventional nature. Given the contrasting reports, the attribution of a causative role to ADMA in the pathogenesis of arterial hypertension in humans remains difficult.

The enzyme dimethylarginine dimethylaminohydrolase (DDAH) (27, 28) is responsible for the hydrolysis of ADMA to citrulline plus methyl-amine, and is encoded by genes *DDAH1* and *DDAH2*. DDAH1 co-localizes with neuronal NOS, whereas DDAH2 can be found in tissues expressing eNOS, such as the endothelium (29). Mendelian randomization is a method that employs genetic polymorphisms known to be able to modify an exposure of interest to determine the existence of a causal association between this exposure (i.e., a biomarker) and a disease. This approach is arguably less prone to reverse causation than other observational studies, because disease status cannot affect germline DNA sequences (30). Most importantly, since genetic variants persist throughout the lifespan, Mendelian randomization studies provide information on life-long exposition to genetically altered levels of biomarkers (in this case, ADMA). The rs9267551 G/C variant in the 5′-untranslated region (UTR) of *DDAH2* has been reportedly demonstrated to affect the quantitative expression of *DDAH2*, specifically in primary human endothelial cells, and a protective role has been proposed for the C allele due to its association with increased NO production (31). Individuals carrying the rs9267551 C allele were shown to have lower circulating levels of ADMA, better response to insulin, and reduced prevalence of chronic kidney disease (31, 32). In a previous work, we reported a significant association with myocardial infarction in two independent cohorts of subjects with type 2 diabetes mellitus, with the C allele consistently showing a protective effect (33). Therefore, we elected to perform a Mendelian randomization study using the functional rs9267551 polymorphism and to assess its effects on *in vivo* measurements of arterial stiffness (by cfPWV), in a heterogenic cohort of subjects, assuming that carriers of the C allele have a vascular protection (as a consequence of higher NO availability).

## Materials and Methods

### Study Subjects

For this study we consecutively recruited 633 adults of European ancestry referred to the Department of Medical and Surgical Sciences of the University “Magna Graecia” of Catanzaro (34). The inclusion criteria were: age ≥ 19 years, and presence of one or more cardio-metabolic risk factors including elevated fasting glucose levels, hypertension, dyslipidemia, overweight/obesity, and family history for diabetes. Exclusion criteria were: end-stage renal disease, chronic gastrointestinal diseases or pancreatitis, history of any malignant disease or of alcohol/drug abuse, hepatic failure or positivity for antibodies to hepatitis C virus (HCV) or hepatitis B surface antigen (HBsAg). Venous blood samples were obtained after a 12-h overnight fast. Body mass index (BMI) was calculated as body weight in kilograms divided by the square of height in meters. Readings of blood pressure (BP) were performed in the non-dominant arm with the patient in supine position, after 5 min of rest, with a sphygmomanometer. Type 2 diabetes was defined according to the American Diabetes Association (ADA) criteria (35). Subjects were classified as hypertensive if they had systolic blood pressure ≥ 130 and/or diastolic ≥ 85 mmHg or in presence of antihypertensive treatment and history of hypertension.

As previously reported (36), we adopted a validated system (Sphygmocor™; AtCor Medical, Sydney, Australia), that utilizes high-fidelity applanation tonometry (Millar) and appropriate software for the analysis of pressure waves (Sphygmocor™). Pressure calibration was obtained with patients lying supine, through automatic recording of brachial BP at the dominant arm, after resting for 30 min (Dinamap Compact T; Johnson & Johnson Medical Ltd, Newport, UK). Measurement of BP was repeated five times, and the average of the final three recordings was used for calibration. Pulse wave was measured at the radial artery of the dominant arm with the wrist softly hyperextended, as the average of single pressure waves during eight consecutive seconds. Pulse wave recordings were admitted if peak and bottom values of single waves showed <5% variability. Aortic pulse wave velocity (PWV) was derived from carotid and femoral pressure waveforms. Carotid to femoral transit time (ΔT) was calculated from the foot-to-foot time difference between carotid and femoral waveforms. The distance between the landmark of the sternal notch and femoral artery was used to estimate the path length between the carotid and femoral arteries (L), and PWV was measured as L/ΔT.

The study was approved by the Local Institutional Ethics Committee of the University “Magna Graecia” of Catanzaro (approval code: 2012.63). Written informed consent was obtained from each subject in accordance with the principles of the Declaration of Helsinki.

### Analytical Determinations

Glucose, triglyceride, total cholesterol and HDL particles concentration was determined by enzymatic methods (Roche, Basel, Switzerland). Plasma insulin concentration was assessed with a chemiluminescence-based assay (Immulite^®^, Siemens, Italy). High performance liquid chromatography with a National Glycohemoglobin Standardization Program certified automated analyzer (Adams HA-8160 HbA1C analyzer, Menarini, Italy) was used to measure HbA1c levels. High sensitivity C reactive protein (hsCRP) levels were determined by an automated instrument (CardioPhase^®^ hsCRP, Milan, Italy). Serum ADMA concentration was measured with Human Asymmetric dimethylarginine (ADMA) ELISA Kit (MBS264847, My BioSource, San Diego, CA, USA). The detection range was 5 μmol/L-0.078 μmol/L, with sensitivity up to 0.01 μmol/L, Intra-assay CV ≤ 8% and inter-assay CV ≤ 12%.

### Genotyping of *DDAH2* Gene Polymorphism

DNA was extracted from whole blood using commercial DNA isolation kits (Promega, Madison, WI and Roche, Mannheim, Germany). rs9267551 *DDAH2* genotype calls were assigned by TaqMan allelic discrimination assay (C__27848488_10; Applied Biosystems, Foster City, CA), after amplification on an iCycler Thermal Cycler with iQ5 Multicolor Real-Time PCR Detection System (Bio-Rad Laboratories, Inc., Hercules, CA). 0.05 ng of custom oligo strings (GeneArt^®^ Strings™ DNA Fragments, Invitrogen, Thermo Fisher Scientific) corresponding to ~200 bp around the context sequence of the genotyping assay, differing only for the rs9267551 allele C or G were loaded onto each plate run to represent one heterozygous C/G and two sets of homozygous C/C and G/G controls. Genotyping concordance of the oligo strings was 100%.

### Statistical Analysis

The estimation of sample size requirements was performed with the program Quanto (version 1.2) and based upon previously reported data obtained in populations similar to ours. To compute our model we assumed an average cfPWV between 6.7 and 7.5 m/s, with a standard deviation (SD) ranging between 1.8 and 2.5 m/s (37, 38), and a minimum minor allele frequency = 4.5% (31–33). In order to be able to detect a clinically relevant difference in cfPWV between genotypes according to a dominant model [at least 1 m/s (13, 39)], with two-sided α = 0.05 and β = 0.80, the recommended sample size was equal to 607 subjects. Each SNP was coded as 0, 1, or 2 depending on the number of C alleles. Therapies were coded as binary variables, 0 indicated absence of treatment, 1, respectively, meant use of anti-hypertensive, glucose lowering agents, or statins as anti-dyslipidemic therapy. Log transformation was employed when analyzing insulin, triglycerides and hsCRP levels because their distribution did not respect the assumption of normality. Comparison of differences between continuous variables in genotype groups were tested by ANCOVA (general linear model) after adjusting for age, sex, and BMI. Categorical variables were compared by χ^2^ test. The Hardy–Weinberg equilibrium between genotypes was evaluated by χ^2^ test. The existing relationships between cfPWV and all collected clinical, biochemical and anthropometrical parameters were explored by univariate regression analysis, with cfPWV as dependent variable. To assess which variables were independently associated with cfPWV we built an exploratory stepwise multivariable linear regression model. The resulting predictive variables were used to compile a predictor factor through principal component analysis. The quality of the reduced-dimension factor was determined through the Bartlett's test for sphercity. The Mediation analysis was conducted through a series of linear regression analyses to calculate the indirect and direct effects and test them for significance (40); we adopted the product of coefficients approach to test the mediating effect of ADMA on the variability of cfPWV, and we tested the significance of the mediation through the Sobel test (41). The significance of indirect and total effects was estimated *via* bootstrapping (40). A two-sided *p-*value < 0.05 was considered statistically significant. All calculations were done with SPSS software program Version 22.0 for Windows.

## Results

A summary of the clinical and anthropometric characteristics of the population, stratified by rs9267551 polymorphism, is reported in Table 1. The study population consisted of 633 unrelated Caucasian subjects (425 men and 208 women with mean age 52 ± 12 years), who were enrolled in the CATAMERI study (42). The genotype distribution of rs9267551 polymorphism was in Hardy–Weinberg equilibrium (*p* > 0.10). GC and CC individuals were collectively considered and analyzed as C carriers, according to a dominant genetic model, because we detected only three individuals with rs9267551 CC homozygous genotype, and because previous functional studies performed in endothelial cells (31) had demonstrated a dominant effect of the C allele. When comparing the two groups (GG vs. GC + CC) the rs9267551 polymorphism did not show any significant association with age, sex, BMI, systolic, and diastolic blood pressure, circulating levels of hsCRP, smoking status, and therapy for hypertension (Table 1). After correction for age, sex and BMI, cfPWV was significantly lower in carriers of the C allele as compared with subjects carrying the GG genotype (6.86 vs. 7.98 m/s, respectively; *P* < 0.01), supporting the hypothesis that constant exposure to higher ADMA levels might lead to reduced vascular elasticity. No differences in lipid profile were observed between groups, despite the higher prevalence of hypolipidemic therapies in the GG group (20.6 vs. 3.8% in the GC + CC group, *P* = 0.01). Similarly, no differences were reported in fasting plasma glucose and insulin levels, although it is worth noticing that the GC + CC group harbored a significantly lower proportion of diabetic subjects (10.3 vs. 29.2% in the GG group, *P* = 0.022) and of subjects undergoing a hypoglycemic regimen (3.8 vs. 15.1% in the GG group, *P* = 0.049).

**Table 1:** Clinical features of 633 study subjects according to the rs9267551 polymorphism of DDAH2.

**Variables**	**Whole cohort**	**GG**	**GC + CC**	***P***
*N*	633	579	54	
Sex (F/M)	208/425	192/387	16/38	0.597
Age (years)	52 (±12)	52 (±13)	50 (±9)	0.299
BMI (Kg/m^2^)	29.34 (±5.1)	29.36 (±5.1)	29.15 (±5.0)	0.764^#^
SBP (mmHg)	137.6 (±16.6)	137.6 (±16.7)	138.3 (±16.1)	0.665^*^
DBP (mmHg)	84.0 (±11.5)	83.7 (±11.6)	86.1 (±10.5)	0.218^*^
Total cholesterol (mg/dl)	199.8 (±38.3)	199.1 (± 38.5)	207.6 (±36.5)	0.132^*^
HDL (mg/dl)	49.0 (±13.6)	49.1 (±13.4)	48.7 (±16.1)	0.932^*^
LDL (mg/dl)	124.0 (±34.1)	123.5 (±34.3)	130.1 (±31.3)	0.209^*^
Triglycerides (mg/dl)	136.7 (±75.1)	136.1 (±75.4)	142.8 (±72.1)	0.508^*^
Fasting glucose (mg/dl)	107.6 (±41.9)	108.4 (±43.2)	99.6 (±24.2)	0.233^*^
Fasting insulin (U/l)	14.2 (±9.7)	14.2 (±9.5)	14.1 (±11.5)	0.944^*^
cfPWv (m/s)	7.88 (±2.79)	7.98 (±2.86)	6.86 (±1.61)	**<0.01** ^*^
hsCRP (mg/L)	3.7 (±4.4)	3.8 (±4.5)	3.4 (±4.1)	0.794^*^
Smoking habit (N/Ex/Y)	353/158/122	324/143/112	29/15/10	0.886
Hypolipidemic therapy (N/Y)	532/101	480/99	52/2	**0.010**
Hypertension therapy (N/Y)	322/311	293/286	29/25	0.663
Diabetes prevalence (N/Y)	497/136	448/131	49/5	**0.022**
Hypoglycemic therapy (N/Y)	555/78	503/76	52/2	**0.049**

*Continuous variables are summarized as means ± SD. Differences of continuous variables between groups were tested after adjusting for age, sex, and BMI by ANCOVA (general linear model). Categorical variables are summarized as absolute number of subjects per category, and compared by χ2 test. A P-value < 0.05 was considered statistically significant and highlighted in bold*.

# *P-values refer to results after adjustment for age and sex*;

* *P-values refer to results after adjustment for age, sex, and BMI. BMI, body mass index; cfPWV, carotid-femoral pulse wave velocity; DBP, diastolic blood pressure; HDL, high density lipoprotein; hsCRP, high sensitivity C-reactive protein; LDL, low density lipoprotein; SBP, systolic blood pressure*.

The association of cfPWV with clinical, biochemical and anthropometrical parameters is reported in Table 2. cfPWV resulted positively and significantly associated with age (β = 0.230, *P* < 0.001), systolic blood pressure (β = 0.152, *P* < 0.001), hsCRP (β = 0.116, *P* < 0.01), fasting plasma glucose (β = 0.133, *P* < 0.001), fasting plasma insulin (β = 0.142, *P* = 0.001), prevalence of diabetes (β = 0.157, *P* < 0.001), and prevalence of treatment with hypoglycemic agents (β = −0.138, *P* < 0.001). On the other hand, circulating HDL levels (β = −0.091, *P* = 0.024) and *DDAH2* rs9267551 C allele (β = −0.110, *P* < 0.01) showed a negative effect on cfPWV, consistent with their protective role exerted on the vasculature. Sex is notoriously associated with cardiovascular risk, but in our population we found that male subjects had only a fringe association with higher cfPWV (β = 0.077, *P* = 0.053). No significant associations were detected between values of cfPWV in our population and BMI, diastolic blood pressure, total cholesterol, LDL particles, circulating triglycerides, prevalence of treatment for hypertension or dyslipidemia, and smoking habit (Table 2).

**Table 2:** Univariate regression analysis with cfPWv as dependent variable.

**Independent contributors**	**β**	***P***
Age (years)	0.230	**<0.001**
Sex (F/M)	0.077	**0.053**
BMI (Kg/m^2^)	0.062	0.122
SBP (mmHg)	0.152	**<0.001**
DBP (mmHg)	0.026	0.511
Total cholesterol (mg/dl)	−0.007	0.856
HDL (mg/dl)	−0.091	**0.024**
LDL (mg/dl)	0.002	0.967
Triglycerides (mg/dl)	0.061	0.128
Fasting glucose (mg/dl)	0.133	**<0.001**
Fasting insulin (U/l)	0.142	**<0.001**
hsCRP (mg/L)	0.116	**<0.01**
Smoking habit (N/Ex/Y)	0.016	0.680
Hypolipidemic therapy (N/Y)	0.067	0.090
Hypertension therapy (N/Y)	0.048	0.229
Diabetes prevalence (N/Y)	0.157	**<0.001**
Diabetes therapy (N/Y)	0.138	**<0.001**
DDAH2 rs9267551 (GG/GC + CC)	−0.110	**<0.01**

*A P-value < 0.05 was considered statistically significant and highlighted in bold. BMI, body mass index; cfPWV, carotid-femoral pulse wave velocity; DBP, diastolic blood pressure; DDAH2, dimethylarginine dimethylaminohydrolase 2; HDL, high density lipoprotein; hsCRP, high sensitivity C-reactive protein; LDL, low density lipoprotein; SBP, systolic blood pressure*.

To estimate the independent contribution of rs9267551 polymorphism to arterial stiffness evaluated through cfPWV, we carried out a multivariable linear regression analysis. The covariates were selected as follows: traditional confounder factors affecting PWV (age, sex, BMI, and smoking habit), variables that resulted significantly associated to cfPWV in our population in the univariate regression analysis mentioned above (SBP, HDL, fasting plasma glucose and insulin, hsCRP, diabetes prevalence and hypoglycemic therapy, Table 2). In order to avoid conflict due to the fact that fasting glucose and insulin levels are strongly correlated to diabetes diagnosis, we built two independent statistical models featuring, respectively, fasting glucose and fasting insulin in Model A and diabetes prevalence in Model B (Table 3). Additionally, when we assessed a third Model, in which treatment with hypoglycemic agents was used as a possible confounding variable in the stead of diabetes prevalence or fasting glucose and insulin levels, we observed no significant influence exerted by the pharmacological treatment on cfPWV (data not shown). The major independent determinants of cfPWV according to Model A were (listed from strongest to weakest): age (β = 0.201, *P* < 0.001), fasting insulin (β = 0.134, *P* < 0.001), systolic blood pressure (β = 0.111, *P* < 0.01) and the rs9267551 polymorphism, with carriers of the C allele having significantly lower vessel rigidity as compared with GG individuals (β = −0.098, *P* = 0.01) (Table 3). Model B revealed a similar pattern of association, with age (β = 0.199, *P* < 0.001), systolic blood pressure (β = 0.105, *P* < 0.01), *DDAH2* rs9267551 genotype (β = −0.100, *P* < 0.01), plus hsCRP (β = 0.078, *P* = 0.044), and HDL (β = −0.076, *P* = 0.049) (Table 3). As a dependent variable, cfPWV did not show any significant association with sex, BMI or smoking habit in neither Model A or B (Table 3).

**Table 3:** Stepwise multiple regression analysis with cfPWv as dependent variable.

	**β**	***P***
**Multivariable regression model A**		
Age (years)	0.201	<0.001
Fasting insulin (mU/ml)	0.134	<0.001
SBP (mmHg)	0.111	<0.01
DDAH2 rs9267551 (GG/GC + CC)	−0.098	0.01
HDL (mg/dl)	0.137	0.939
Smoking habit (N/Ex/Y)	0.985	0.989
BMI (Kg/m^2^)	0.727	0.876
Fasting glucose (mg/dl)	0.307	0.883
Sex (F/M)	0.147	0.982
hsCRP (mg/L)	0.050	0.975
**Multivariable regression model B**		
Age (years)	0.199	<0.001
SBP (mmHg)	0.105	<0.01
DDAH2 rs9267551 (GG/GC + CC)	−0.100	<0.01
hsCRP (mg/L)	0.078	0.044
HDL (mg/dl)	−0.076	0.049
Smoking habit (N/Ex/Y)	0.780	0.975
BMI (Kg/m^2^)	0.552	0.883
Sex (F/M)	0.186	0.856
Diabetes prevalence (N/Y)	0.118	0.840

*Model A includes as covariates: age, fasting insulin, SBP, DDAH2 rs9267551 genotype, HDL, fasting glucose, sex, BMI, smoking habit, and hsCRP. In Model B fasting glucose and insulin levels have been replaced by diabetes prevalence. BMI, body mass index; cfPWV, carotid-femoral pulse wave velocity; DDAH2, dimethylarginine dimethylaminohydrolase 2; HDL, high density lipoprotein; hsCRP, high sensitivity C-reactive protein; SBP, systolic blood pressure*.

**Figure 1:**
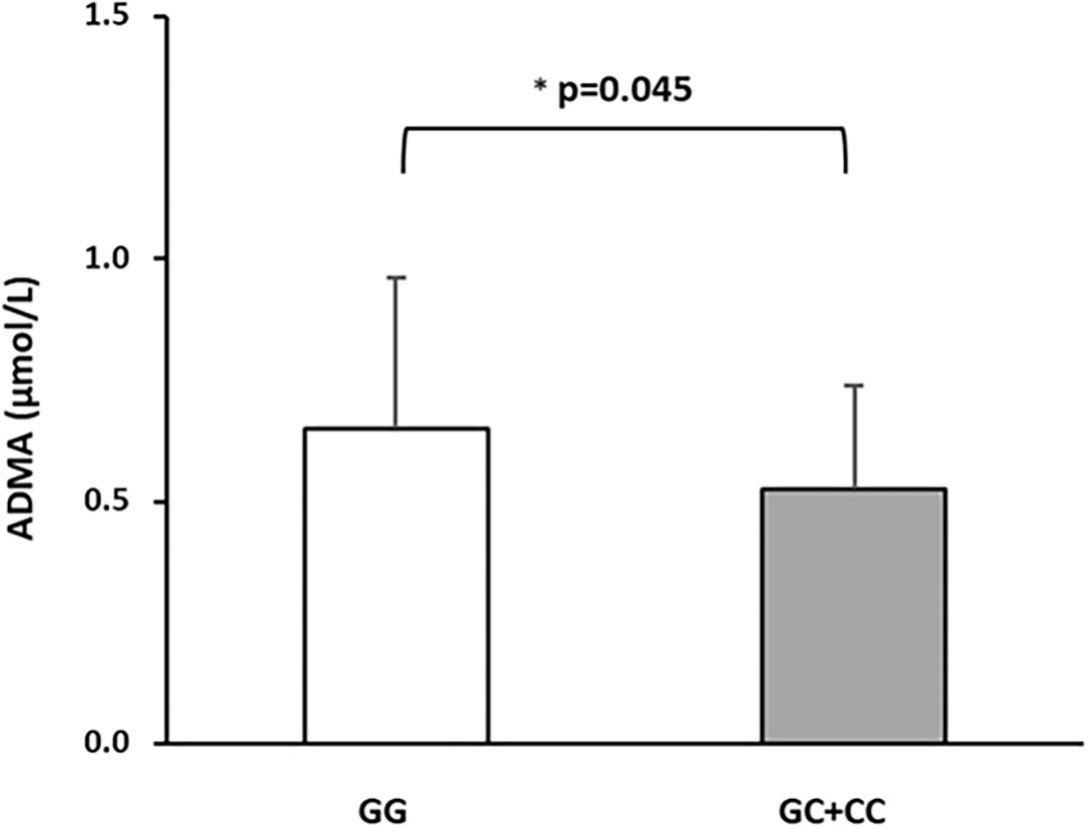
Differences in circulating ADMA concentrations in a subset of 344 subjects stratified based on DDAH2 rs9267551 genotypes. The white bar represents the rs9267551 GG genotype (*n* = 321) and the grey bar represents the rs9267551 GC + CC genotype (21 subjects with GC genotype and 2 with CC homozygous). ^*^p-value after correction of age, sex, and BMI. Data are means ± Standard Deviation.

In order to exclude any confounding influence deriving from other factors associated with *DDAH2* rs9267551 genotype (by chance, or pleiotropy), we constructed a final multivariable regression model (Supplementary Table) encompassing traditional risk factors for arterial stiffness and covariates which resulted associated either with cfPWV, as in Model B, plus the remaining variables associated to rs9267551 in our population (hypolypidemic therapy, Table 1). The independent contribution of each trait to the variability of cfPWV resulting from this supplementary model was almost superimposable to the results obtained from Model B. Serum ADMA concentrations were assessed for a subset of 344 subjects whose biological specimen were available. In this subset there were 321 GG genotypes, and 23 carriers of the C allele (21 subjects with GC genotype and 2 CC homozygous). Consistent with previous data, circulating ADMA was higher in GG individuals than in C allele carriers (0.65 ± 0.31 vs. 0.52 ± 0.21 μmol/l, respectively; *P* = 0.045 after correction for age, sex, and BMI, Figure 1). At first, we elected to replicate the analysis as in multivariable regression Model B in order to ensure that the subset was a *bona fide* representative of the full cohort, and we confirmed that the observed genetic effect was preserved (Model C, Table 4) with *DDAH2* rs9267551 C allele exerting a comparable protective action (β = −0.102, *P* = 0.04) over cfPWV. When we added the measurement of serum ADMA levels to the statistical model (Model D, Table 4), it manifested a stronger independent effect on cfPWV (β = 0.139, *P* = 0.006), and the contribution of *DDAH2* rs9267551 genotype became non-significant (β = 0.088, *P* = 0.969).

**Table 4:** Stepwise multiple regression analysis in the subset sample, with cfPWv as dependent variable.

	**β**	***P***
**Subset analysis model C**		
hsCRP (mg/L)	0.189	<0.0001
Age (years)	0.183	<0.001
SBP (mmHg)	0.175	<0.01
Diabetes prevalence (N/Y)	0.155	<0.01
DDAH2 rs9267551 (GG/GC + CC)	−0.102	<0.04
Sex (F/M)	0.171	0.159
HDL (mg/dl)	−0.070	0.164
Smoking habit (N/Ex/Y)	0.027	0.600
BMI (Kg/m^2^)	0.001	0.986
**Subset analysis Model D**		
Age (years)	0.172	0.02
hsCRP (mg/L)	0.167	0.001
SBP (mmHg)	0.166	0.001
Diabetes prevalence (N/Y)	0.164	0.003
ADMA (μmol/L)	0.139	0.006
DDAH2 rs9267551 (GG/GC+CC)	−0.088	0.969
Sex (F/M)	0.075	0.131
HDL (mg/dl)	−0.062	0.076
Smoking habit (N/Ex/Y)	0.019	0.712
BMI (Kg/m2)	0.001	0.978

*Model C includes as covariates: age, SBP, diabetes prevalence, DDAH2 rs9267551 genotype, HDL, sex, BMI, smoking habit, and hsCRP. In Model D serum ADMA levels are added to the covariates in Model C. BMI, body mass index; cfPWV, carotid-femoral pulse wave velocity; DDAH2, dimethylarginine dimethylaminohydrolase 2; HDL, high density lipoprotein; hsCRP, high sensitivity C-reactive protein; SBP, systolic blood pressure*.

**Figure 2:**
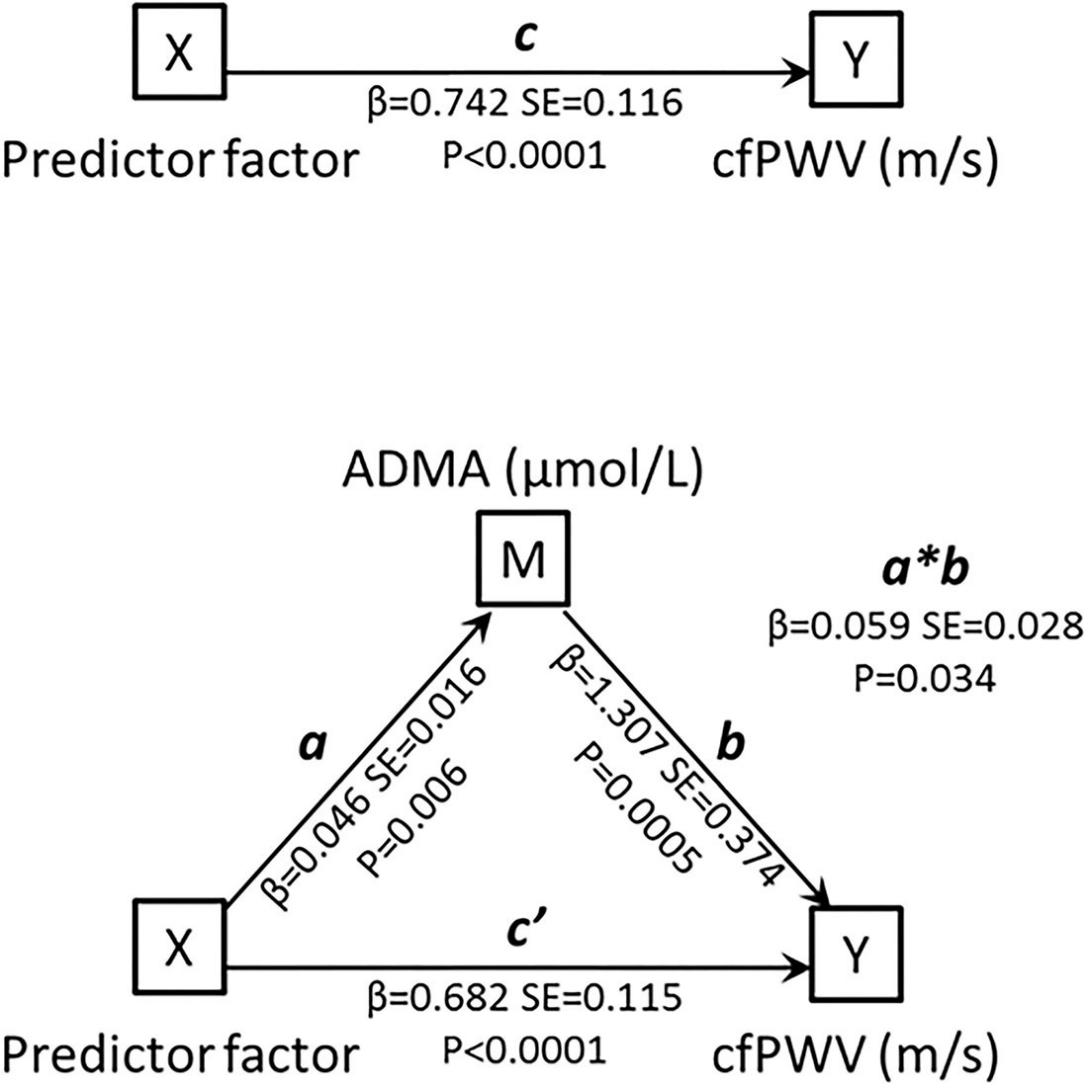
Schematic representation of the mediation model. The mediation analyses were conducted using a series of linear regression models to estimate the total effect of X on Y *(c)*, the indirect effect of X on Y (a^*^b), and the direct effect of X on Y with removal of the effect of the mediator (c'). M, mediating variable; X, independent variable; Y, dependent variable.

This observation prompted us to the performance of a mediation analysis, graphically summarized in Figure 2. As predictor variable we adopted a factor produced by principal component analysis encompassing age, hsCRP, SBP, diagnosis of diabetes and *DDAH2* rs9267551 genotype (χ^2^ = 90.8, *P* < 0.0001). This predictor factor had eigenvalue = 1.490 and explained 29.806% of total variance. The total effect of the predictor factor on cfPWV, including the indirect and direct effect, is represented by the coefficient *c* (β = 0.742, SE = 0.116, *P* < 0.00001). The coefficient *a* (β = 0.046, SE = 0.016, *P* = 0.006) represents the effect of the predictor factor on ADMA, and the coefficient *b* (β = 1.307, SE = 0.374, *P* = 0.0005) represents the effect of ADMA on cfPWV. The coefficient *c'* (β = 0.682, SE = 0.115, *P* < 0.00001) represents the direct effect of the predictor factor on cfPWV, after correcting for the effect of ADMA. Finally, the mediated (or indirect) effect of the predictor factor on cfPWV through ADMA is represented as a^*^b (β = 0.059, SE = 0.028), and it was statistically significant *via* the bootstrapped estimation approach of the Sobel test, (*P* = 0.034). Thus, as both the direct and the mediated effects between the predictor factor and cfPWV were statistically significant, it may be suggested that a partial mediation exists, and this hypothesis is further supported by the fact that the direct effect *c'* has a smaller magnitude than the total effect *c*. Because *DDAH2* polymorphism rs9267551 was the only parameter included in the predictor factor to lose its association with cfPWV when circulating ADMA levels were added in the multivariable regression Model D, it is possible to infer that the effect of rs9267551 genotype on cfPWV is mediated by ADMA.

## Discussion

In the current study, we report, for the first time, evidences hinting that the presence of rs9267551 C allele may protect, at least in part, from an increase in arterial stiffness as measured by cfPWV, independently from the presence of traditional cardiovascular risk factors including age, sex, BMI, smoking habit and type 2 diabetes. Furthermore, our observations support the attribution of a causative role to ADMA in the pathogenesis of arterial rigidity, in line with evidences that long-term ADMA infusion is able to induce arteriosclerotic damage in an animal model of eNOS deficient mice (43), and that the acute infusion of subpressor doses of ADMA (0.10 mg/kg/min) increases vascular stiffness and decreases cerebral perfusion in healthy humans, independently from changes in blood pressure (25).

The measurement of PWV is a simple, non-invasive, and trustworthy method to evaluate the rigidity of a segment in the arterial system, and its use is advised by several guidelines (8, 10). More specifically, cfPWV is considered the gold-standard method for the measurement of aortic stiffness in a simple and non-invasive way (44). Several studies have shown that high cfPWV predict the occurrence of cardiovascular diseases even more than other traditional major risk factors (2, 6, 11, 12, 45, 46). Our population showed a difference in cfPWV of more than 1 m/s when the groups based on rs9267551 polymorphism were compared (Table 1). This difference is clinically relevant, as discussed in 2010 by the meta-analysis authored by Vlachopoulos et al. (13) that evaluated 15,877 ethnically etherogenous subjects with variable high baseline risk (populations with hypertension, diabetes, end-stage renal disease, coronary artery disease, and subjects from the general population). The results of the meta-analysis revealed that the relative risk associated with an increase of 1 m/s in aortic PWV was 1.14 (95% CI: 1.09-1.20) for total CV events, 1.15 (95% CI: 1.09-1.21) for cardiovascular mortality, and 1.15 (95% CI: 1.09-1.21) for all-cause mortality.

ADMA is an endogenous inhibitor of eNOS activity, it competes for the binding of eNOS active site with L-Arginine, which is the substrate for the synthesis of NO. There is substantial evidence that NO bioavailability is associated with the levels of various components of the L-arginine/NO pathway (47), and several studies have documented a significant association between ADMA and hypertension (48–50). Furthermore, increased levels of ADMA are considered predictors of cardiovascular morbidity and mortality (18) and have been proposed as an explanation for the reduced production of NO typically observed in insulin resistance (50–52). Hence, serum ADMA levels have the potential to serve as diagnostic markers and therapeutic targets, however, animal and human studies have supplied conflicting results, and could not ultimately determine whether ADMA has a causal role in the pathogenesis of arterial stiffness and hypertension (26, 53, 54).

The hydrolysis of ADMA catalyzed by the DDAH enzyme is a major determinant of plasma ADMA concentration (27). Notably, animal models in which the modulation of ADMA had been constricted through overexpression or knockout of *DDAH* have provided support to the hypothesis that ADMA might play a pathogenic role (53–55), whereas other models have failed to report consistent evidences (56–58). This discrepancy suggests that pharmacological lowering of ADMA by targeting DDAH could be the key to understand whether ADMA directly plays a role in human cardiovascular pathology.

Demonstrating the existence of a causal role for ADMA in vessel dysfunction has the potential to determine meaningful changes in the clinical practice, and few therapeutic approaches specifically targeting ADMA pathways have already been proposed (59, 60). Among them, there is one experimental molecule with DDAH properties (M-DDAH) that resulted effective at lowering ADMA in preclinical models (61). Therefore, it is important to clarify the pathological role of ADMA in humans, in order to set the foundation in support of future studies. To achieve this objective we applied a Mendelian randomization study design, with the final goal of understanding if ADMA levels are associated with higher vascular stiffness, measured through cfPWV. This approach requires that the selected instrument (in this case SNP rs9267551) associates with the exposure. In our study, ADMA is the exposure and, although previous researches evaluating the impact of genetic variation of the *DDAH2* locus with cardiovascular risk have not supplied consistent results (62–64), the rs9267551 variant in the 5′-UTR of *DDAH2* has been proven to have a robust functional impact: primary human endothelial cells obtained from donors carrying the C allele showed increased transcription of *DDAH2*; and circulating ADMA levels were reduced in adult human subjects carrying the C allele when compared to the rs9267551 GG homozygous group (31). These evidences suggest that the rs9267551 C polymorphism in the locus of *DDAH2* may protect eNOS activity and NO production through its effects on ADMA levels. Indeed, in a previous published paper, we reported a role for our instrument, the functional polymorphism rs9267551, in modulating the risk of myocardial infarction even among diabetic patients (33). To date, no data are available in the literature about the impact of polymorphism rs9267551 on arterial stiffness.

Our results support the hypothesis that rs9267551 genotype may affect cfPWV, and suggest that this effect is mediated by circulating ADMA levels. In addition to this, it is worth noticing that the association between rs9267551 and cfPWV was independent from blood pressure, an observation that, if confirmed, might help elucidate, at least partially, the discrepancies in the literature about the causal role of ADMA in the pathogenesis of arterial stiffness and hypertension. Indeed, our data hint that long-term exposure to higher ADMA levels determines a specific vascular risk *via* the alteration of vessel elasticity (as measured by cfPWV) without affecting arterial hypertension.

The present study has some strengths including the exclusion of possible biases deriving from the presence of infectious or malignant conditions by study design, the accurate characterization of all enrolled subjects (no self-reported data were used) that permitted to encompass multiple recognized cardio-metabolic risk factors in the analyses, and the compliance with the assumptions of Mendelian randomization. Moreover, the homogeneity of the ethnicity of the population (all Italians of European descent), the accuracy of data collection via standardized protocol, and the centralized laboratory determinations, represent other points of strength of our analysis.

Notwithstanding this, several limitations are also present. First, the cross-sectional nature of the design does not permit to evaluate incident vascular dysfunction; therefore the present results require replication in prospective studies for validation. Furthermore, unfortunately, due to the low minor allele frequency, we were not able to establish the effect relative to allelic dosage (“per allele” effect), and biological specimen for laboratory determination of circulating ADMA levels were only available for a subset of our population. However, we have been able to confirm our previous report that ADMA levels were significantly higher in individuals with rs9267551 GG genotype than in C allele carriers (31). Although the robustness of our main claim (the association between rs9267551 genotype and cfPWV) is significantly reinforced by the positive independent linear relationship between circulating ADMA levels and cfPWV, we cannot completely exclude the influence of residual confounding. Additionally, caution should be exerted in extending the present findings to the general population, because they were based upon observational data collected from Caucasian subjects of European ancestry with mild to high cardiometabolic risk.

In conclusion, to the best of our knowledge, we are supplying the first evidences in support of the hypothesis that the variant rs9267551 in the *DDAH2* gene can modulate cfPWV in Italian adult subjects, and we have been able to demonstrate the existence of an interaction between rs9267551 genotype and circulating ADMA levels, able to affect arterial stiffness. Hopefully, replication of these results in future studies will allow their extension to other populations of different ethnic background, with the final goal of definitively unraveling the role of ADMA in the pathogenesis of vascular risk and if intervening on *DDAH2* may turn out to be a fruitful therapeutic/preventive strategy.

## Data Availability Statement

The original contributions presented in the study are included in the article/Supplementary Material, further inquiries can be directed to the corresponding author/s.

## Ethics Statement

The study was approved by the Institutional Ethics Committee of the University Magna Graecia of Catanzaro (Approval Code: 2012.63). Written informed consent was obtained from each subject in accordance with the principles of the Declaration of Helsinki.

## Author Contributions

GM, CA, and EM researched data, reviewed, and edited the article. EM, RS, SM, TF, GM, ES, and AS researched data. GM, PJ, and GS contributed to the discussion and reviewed the article. GM and GS analyzed the data and reviewed the article. FA designed the study, analyzed the data, and wrote the article. All authors contributed to the article and approved the submitted version.

## Funding

GM was supported by funds from the EU project AIM1829805-3.

## Conflict of Interest

The authors declare that the research was conducted in the absence of any commercial or financial relationships that could be construed as a potential conflict of interest.

## Publisher's Note

All claims expressed in this article are solely those of the authors and do not necessarily represent those of their affiliated organizations, or those of the publisher, the editors and the reviewers. Any product that may be evaluated in this article, or claim that may be made by its manufacturer, is not guaranteed or endorsed by the publisher.
