# Supplementary material for: The Functional Polymorphism of DDAH2 rs9267551 Is an Independent Determinant of Arterial Stiffness
Source: Front Cardiovasc Med. 2022 Jan 3;8:811431. doi: 10.3389/fcvm.2021.811431 (PMC8761764; doi:10.3389/fcvm.2021.811431)
Supplement: Supplementary file 1 [file Table_1.DOCX]

Supplementary Material

# Supplementary Table

| **Final multivariable regression model** | **β** | **P** |
| --- | --- | --- |
| **Age (years)** | 0.199 | <0.001 |
| **SBP (mmHg)** | 0.105 | <0.01 |
| **DDAH2 rs9267551 (GG/CG+CC)** | -0.100 | <0.01 |
| **hsCRP (mg/L)** | 0.078 | 0.044 |
| **HDL (mg/dl)** | -0.076 | 0.049 |
| Smoking habit (N/Ex/Y) | 0.813 | 0.975 |
| Hypolipidemic Therapy (N/Y) | 0.992 | 0.900 |
| Gender (F/M) | 0.186 | 0.856 |
| BMI (Kg/m^2^) | 0.552 | 0.883 |
| Diabetes prevalence (N/Y) | 0.118 | 0.840 |

**Supplementary Table. Stepwise multiple regression analysis with cfPWV as dependent variable.** BMI = body mass index; cfPWV = carotid-femoral pulse wave velocity; *DDAH2* = *dimethylarginine dimethylaminohydrolase 2*; hsCRP = high sensitivity C-reactive protein; HDL = high density lipoprotein; SBP = systolic blood pressure.
